# Malaysia and COVID-19: In Data We Trust

**DOI:** 10.21315/mjms2020.27.6.1

**Published:** 2020-12-29

**Authors:** Kamarul Imran Musa, Jafri Malin Abdullah

**Affiliations:** 1 Editorial Board Member, Malaysian Journal of Medical Sciences, School of Medical Sciences, Universiti Sains Malaysia, Kelantan, Malaysia; 2 Department of Community Medicine, School of Medical Sciences, Universiti Sains Malaysia, Kelantan, Malaysia; 3 Brain and Behaviour Cluster, School of Medical Sciences, Universiti Sains Malaysia, Kelantan, Malaysia

**Keywords:** COVID-19, SARS-Cov-2, non-pharmacological interventions, surveillance

## Abstract

The recent spike of transmissibility of COVID-19 was evident by a large number of COVID-19 cases and apparent quick spread of SARS-CoV-2 in the state of Sabah, Selangor and Negeri Sembilan in Malaysia. The question remains as to what are the main contributory factors for the impending COVID-19 second wave in Malaysia and why the current surveillance system fails to show signs of the impending second — or the third — COVID-19 wave. In public health surveillance, data are the ultimate indicator, and in the era of big data and the Industrial Revolution 4.0, data has become a valuable commodity. The COVID-19 data keeper must fulfil some criteria to ensure COVID-19 data are useful. Researchers are obligated to share their COVID-19 data responsibly. The surveillance for COVID-19 is paramount, and the guidelines such as the one published by the World Health Organization ‘Public health surveillance for COVID-19: interim guidance’ must be referred to. Data must be taken seriously and shared to enable scientists, clinicians, epidemiologists and public health experts fight COVID-19.

Malaysia is experiencing another significant increase in new confirmed COVID-19 cases. This time, the situation is worse than the first wave of COVID-19 seen between March and June 2020. On 8 August 2020, there were 62 new confirmed cases; however, following that, the daily confirmed cases increased exponentially until early November 2020. Currently, the numbers are hovering around 1000 cases per day (Figures 1 and 2).

Some epidemiologists consider the current spike of COVID-19 cases as the second wave of COVID-19. Others see this as the third wave. At the beginning of the second wave — or the third wave for others — the transmissibility of COVID-19 was evident by a large number of COVID-19 cases and apparent quick spread of SARS-CoV-2 in the state of Sabah in Borneo (1). However, in the last week or so, a few states in Peninsular Malaysia, namely Selangor and Negeri Sembilan, contributed a significant proportion of the new confirmed COVID-19 cases (2, 3).

The question remains as to what are the main contributory factors for the impending COVID-19 second wave in Malaysia. Epidemiologists would ask if they have missed identifying its signs. At the beginning of July 2020, there were 8,639 cumulative confirmed COVID-19 cases nationwide. This number jumped to 70,236 on 4 December 2020. Based on the numbers of confirmed COVID-19 cases, the evidence is clear that the transmissibility of COVID-19 has increased. Should we have picked up on the impending second wave earlier? Did our publicly available data — updated daily by the Ministry of Health — fail to show signs of the increasing transmission of SARS-CoV-2? Data monitoring should act as the early warning system for public health surveillance, and if these data fail to do so, then the monitoring system must be improved.

In public health surveillance, data are the ultimate indicator. Especially in the era of big data and the Industrial Revolution 4.0, data has become as valuable as oil, if not more so, because it contains information used to understand current problems. Scientists use data to develop predictive models of human behaviours. Data is so powerful that human behaviours can be influenced by it (4, 5). Data’s role in the US election and the major ethical issue with Cambridge Analytica are two examples of data’s significant value.

In pandemic situations, data becomes more critical. Since the start of the COVID-19 pandemic in early November 2019, we have seen a significant increase in the number of dashboards tracking COVID-19 cases. Some go the extra mile by incorporating predictive capabilities in their engines to make projections about the spread and impact of COVID-19. A few dashboards that have become the points of reference for scientists globally include the Johns Hopkins Coronavirus Resource Center (https://coronavirus.jhu.edu/map.html) (6), the coronavirus pandemic (COVID-19) (https://ourworldindata.org/coronavirus) (7) and the COVID-19 projections (https://covid19.healthdata.org/global) (8).

As the pandemic progresses, consistent measurement of its scale across time and space should be a priority. Gathering objective and comparable data is crucial to determine the effectiveness of different national strategies used to mitigate and suppress, and thus to better prepare for the probable continuation of the epidemic over the next year or more. Disseminating this information should be done within 3–4 weeks of the observation period, and it should be shared at the international level for easier and more accurate comparisons (9, 10). At a minimum, tabulations by sex and 5-year age groups are essential. Where not already in the public domain, countries should also release the equivalent weekly data for every calendar year from 2010 for the calculation of excess deaths in 2020 (9).

Most, if not all, countries have mandated the keeping of COVID-19 data. Data will be reported based on a few parameters such as the confirmed COVID-19 cases, number of contact tracings and results from laboratory tests. From these parameters, the COVID-19’s time trend data will then be displayed. In most countries, the sharing will be nationwide, using television networks and social media. We believe this is commendable as sharing adequate data with the public and researchers will help us to understand the transmission dynamics better. This understanding would later be translated into suppression or intervention strategies, such as mitigation actions of COVID-19. The data can be compared to quantify the effects of interventions and behavioural changes. In our article published in the early phase of the pandemic, we encouraged data sharing to combat COVID-19 (11). Unfortunately, the sharing of good quality COVID-19 data in the local context of Malaysia up to now is almost non-existent.

We believe for data to be useful for COVID-19 researchers, the data keeper must fulfil these criteria:

- Data must be as complete as possible, as determined by the analytical objectives.
- Because COVID-19 spreads very quickly, data must be logged and shared as quickly as possible so that results can be produced as soon as possible.
- Collection of data and definition of variables in the data must be transparent and ethically approved. This can avoid misleading and malicious findings; for example, some COVID-19 articles have been retracted from *The Lancet* and the *New England Journal of Medicine* (12).
- Data should be organised and easily shared. Preferably, data should be available in tabular formats, such as comma-separated values easily downloadable from the data.world website (https://data.world/), GitHub (https://github.com/) or the Ministry of Health Malaysia’s official COVID-19 resource centre.
- Responsible agencies must vet the data to ensure reliability and validity.

Similarly, researchers are obligated to share their COVID-19 data responsibly. This could be done through these measures:

- The government must make it mandatory for researchers to share their data. This is already happening in some parts of the world. The Wellcome Trust and the Bill & Melinda Gates Foundation mandated that funding recipients share data from research related to COVID-19 (13).
- Researchers must provide a list of their COVID-19 research projects and links to access and download the data.
- Researchers must provide a COVID-19 data dictionary and adequate metadata.
- Researchers must document the data-wrangling process so that it is reproducible.
- Researchers must indicate to the human ethics committee that their COVID-19 data will be shared and will not be destroyed within a specific time.

Another area where data is essential is COVID-19 surveillance. The World Health Organization has published a document titled ‘Public health surveillance for COVID-19: interim guidance’ (14). The general objective of COVID-19 surveillance is to enable public health authorities to reduce the transmission of COVID-19, thereby limiting associated morbidity and mortality. Specifically, data from COVID-19 surveillance will enable epidemiologists, physicians and health workers to: i) enable rapid detection, isolation, testing and management of cases; ii) monitor trends in COVID-19 deaths; iii) identify, follow up with and quarantine contacts; iv) detect and contain clusters and outbreaks, especially among vulnerable populations; v) guide the implementation and adjustment of targeted control measures while enabling the safe resumption of economic and social activities; vi) evaluate the pandemic’s impact on healthcare systems and society; vii) monitor longer-term epidemiologic trends and the evolution of the SARS-CoV-2; and virus; and viii) contribute to the understanding of the co-circulation of the SARS-CoV-2 virus, influenza and other respiratory viruses and pathogens.

Data is fundamental to everyday life. For the public, data can help people to adjust their behaviours to minimise the risk of spreading SARS-CoV-2. For health professionals, sharing COVID-19 data helps them to quantify the impact of non-pharmacological interventions (NPI). For COVID-19 researchers, data is paramount to understand the infection dynamics, make projections and identify the high-risk populations. Due to the Industrial Revolution 4.0, the world is connected with high-speed data and the rise of data science; data is critical every second of every day. Data must be taken seriously and must be shared with skilled people who can harness it, including every COVID-19 scientist, clinician, epidemiologist and public health expert for them to fight COVID-19. At the national level, Malaysia launched the Malaysian Government Central Data Exchange (MyGDX) platform in 2018. If the government of Malaysia enables this platform for the sharing of COVID-19, we could be one of the countries at the forefront of COVID-19 data sharing.

## Figures and Tables

**Figure 1:**
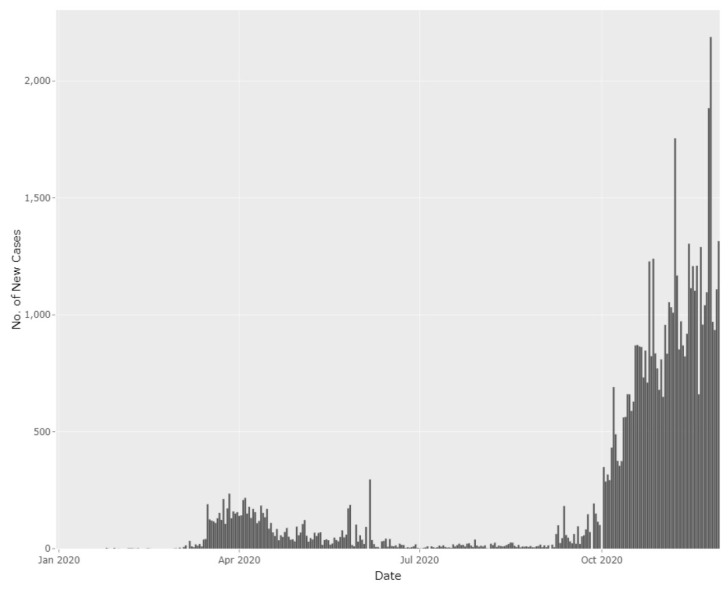
The epidemic curve based on the number of daily confirmed COVID-19 cases in Malaysia

**Figure 2:**
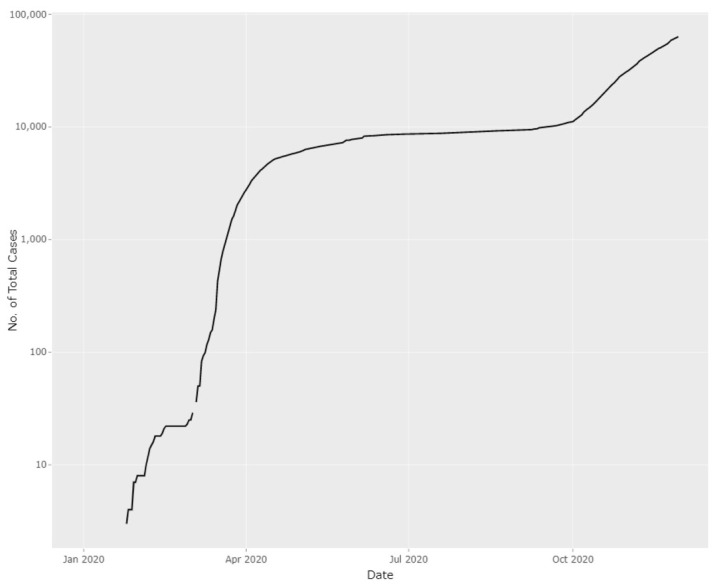
The cumulative number of confirmed COVID-19 cases in Malaysia on a log scale
